# MicroRNA-Integrated and Network-Embedded Gene Selection with Diffusion Distance

**DOI:** 10.1371/journal.pone.0013748

**Published:** 2010-10-29

**Authors:** Di Huang, Xiaobo Zhou, Christopher J. Lyon, Willa A. Hsueh, Stephen T. C. Wong

**Affiliations:** 1 Bioinformatics Core, The Methodist Hospital Research Institute, Weill Medical College, Cornell University, Houston, Texas, United States of America; 2 Diabetes Research Center, The Methodist Hospital Research Institute, Weill Medical College, Cornell University, Houston, Texas, United States of America; Fondazione Telethon, Italy

## Abstract

Gene network information has been used to improve gene selection in microarray-based studies by selecting marker genes based both on their expression and the coordinate expression of genes within their gene network under a given condition. Here we propose a new network-embedded gene selection model. In this model, we first address the limitations of microarray data. Microarray data, although widely used for gene selection, measures only mRNA abundance, which does not always reflect the ultimate gene phenotype, since it does not account for post-transcriptional effects. To overcome this important (critical in certain cases) but ignored-in-almost-all-existing-studies limitation, we design a new strategy to integrate together microarray data with the information of microRNA, the major post-transcriptional regulatory factor. We also handle the challenges led by gene collaboration mechanism. To incorporate the biological facts that genes without direct interactions may work closely due to signal transduction and that two genes may be functionally connected through multi paths, we adopt the concept of diffusion distance. This concept permits us to simulate biological signal propagation and therefore to estimate the collaboration probability for all gene pairs, directly or indirectly-connected, according to multi paths connecting them. We demonstrate, using type 2 diabetes (DM2) as an example, that the proposed strategies can enhance the identification of functional gene partners, which is the key issue in a network-embedded gene selection model. More importantly, we show that our gene selection model outperforms related ones. Genes selected by our model 1) have improved classification capability; 2) agree with biological evidence of DM2-association; and 3) are involved in many well-known DM2-associated pathways.

## Introduction

A fundamental goal of modern biology is to understand complex biological phenomena at the molecular level. In most cases, the first step towards achieving this goal is to isolate genes that are important to a particular biological process. Various statistical concepts and machine learning models have been used to identify genes in microarray data that reveal differential expression patterns according to a selected phenotype status, including *t*-, *F*- and Wilcoxon statistics and signal-noise-ratio [1], mutual information [2], support vector machine [3], Bayesian network [4], and random forest [5] approaches. Strategies have also been proposed to address different issues, such as noisy data and small sample size [6], [7], [8]. More recently, gene interaction information has been introduced into the gene selection (GS) process to account for the known ability of genes to interact or react within pathways or common modules. These approaches first identify differentially-expressed genes within microarray data, and then evaluate the performance of pre-defined functional gene categories (or networks) to identify the genes that associate with the phenotype of interest (see [9] for a comprehensive review of these approaches). Gene functional category or network information can improve the sensitivity of GS, detecting useful genes that only present subtle expression changes among different assayed phenotypes. For example, in a pioneering study, gene set enrichment analysis (GSEA) was used to identify a set of oxidative phosphorylation-related genes that modestly but coordinately decrease in skeletal muscle of diabetic human subjects. Researchers have since further modified this type of analysis by incorporating the topology of gene networks. Rahnenführer et al. [10] have emphasized gene pairs with little network distance to score the cooperativeness of a gene network. Rapaport et al. [11], after assuming that genes and their network neighbors should show similar expression levels, have used a spectral-graph-based method to reduce microarray data noise in order to enhance classification accuracy. More recently, Wei and Li [12] designed a Markov random field model to evaluate genes based on both their own expression and the expression of their directly-connected neighbors. The results from all these studies demonstrate that analyzing the topology of gene networks can improve GS and classification.

Microarray values represent the levels of gene transcripts (i.e., mRNAs), and are routinely used as direct surrogates for gene expression in most GS methods developed to date, including all the methods cited above. This requires the implicit assumption that gene expression directly mirrors gene transcript levels. This assumption appears to be acceptable since it is consistent with biological observation in most cases [13]. Direct overlay of mRNA expression levels onto gene networks can therefore work well and produce promising results. However, improvement is still needed, since post-transcriptional processes have been widely reported to impact gene expression levels, sometimes to great effect.

MicroRNA expression has recently been shown to be a very important means of posttranscriptional regulation of gene expression. MicroRNAs regulate metazoan gene expression by complementarily binding to frequently-imperfect recognition sequences that are predominantly located within the 3′ untranslated regions (UTR) of a target mRNA, with few exceptions [14], [15]. MicroRNAs can regulate the expression of their target genes at multiple levels, blocking mRNA translation and/or inducing mRNA degradation [16], and have been suggested to play roles in a broad range of biological processes, such as developmental timing, cell growth and differentiation, apoptosis and cell proliferation. Furthermore, accumulating evidence implies that microRNAs are associated with bio-molecular characteristics of various human complex diseases, such as cancer [17], [18] and diabetes [19], [20]. With these observations, and supported by the advances already made in the study of microRNA, it is necessary and possible for a GS model to address potential regulatory microRNA effects.

We herein propose a microRNA-integrated network-embedded gene selection (MiNeGS) model to investigate the ability of microRNA information to enhance GS. Our model has two major contributions. First, microarray data and microRNA binding information are integrated prior to evaluation of gene correlation, since mRNA expression level and microRNA-mediated post-transcriptional regulation are known to be key factors influencing gene expression. We propose that two genes are closely correlated when their mRNA levels vary coordinately in the face of similar potential microRNA regulation on them. Gene transcript levels are directly derived from microarray data, while microRNA regulatory effects are modeled using mRNA sequence information, with the assumption that mRNA with similar putative microRNA binding sites are likely to experience similar posttranscriptional regulation. Second, we introduce the concept of diffusion distance to detect the nearest functional partners (NFPs) of a gene. Genes generally interact in highly complicated patterns. Genes, either having direct or indirect network connections to each other, may function closely [21], through signal transduction. Also the signal transduction from one gene to another can be done through multiple paths, instead of a single one. We therefore utilize the concepts of diffuse distance and diffuse maps [22] in order to detect both directly- and indirectly-interacting NFPs as well as to capture potential multi-path connections between genes.

## Results and Discussion

### A. Overview of the proposed MiNeGS model

**Figure 1:**
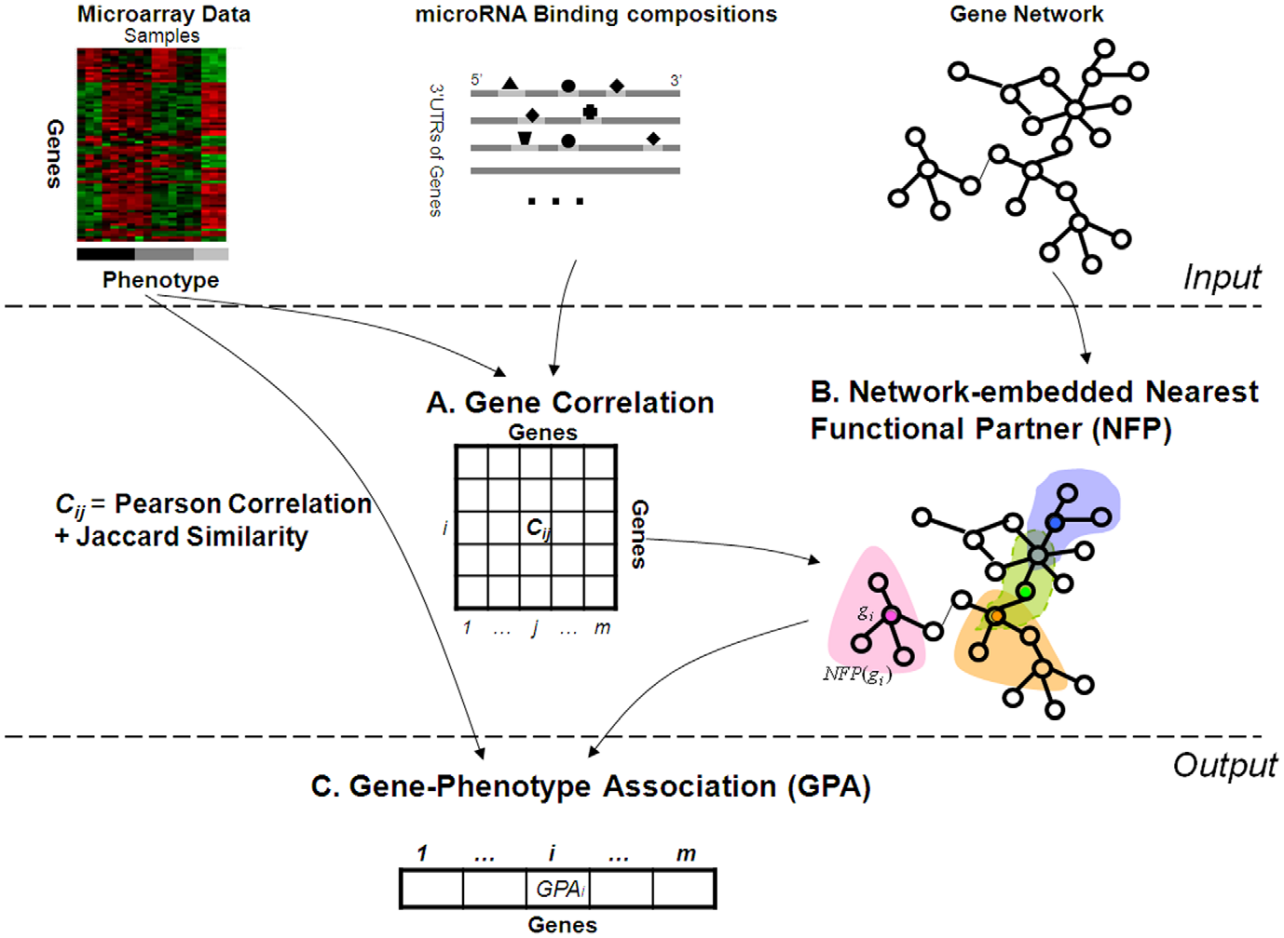
Outline of the proposed method.

Our microRNA-integrated network-embedded gene selection model (MiNeGS) includes three components (A, B and C), as depicted in Fig. 1 and further described in Materials and Methods. Briefly, in Part A, the expression similarity between two genes is calculated based on two factors: their steady-state mRNA expression levels, recorded by microarray data, and their potential from microRNA-mediated posttranscriptional regulation, as indicated by their microRNA binding sites. In Part B, gene correlations estimated in Part A are used to weight the edges of our gene network. Based on this weighted gene network, the functional distance of any two genes are evaluated using the concept of diffusion distance, and the genes with the *k* smallest functional distance to gene *g* are selected as its nearest functional partners (NFPs). Small *k* values prevent the exploitation of network information, restricting the contributions of our network-based GS strategy, while large *k* values may link genes with NFPs that do not share the same functions or fall within the same pathways. In this study, we set *k* with a modest value, *k* = 3, to avoid over- and under-identification of NFPs. Finally, in Part C, gene-phenotype associations are estimated for all genes by averaging the expression-based correlations of genes and their NFPs. Computational analyses performed using these three components allow genes with high phenotype correlations to be selected from the dataset. Our major contributions lie in the development of a method to integrate gene expression and microRNA binding information (Part A) and a diffusion-distance-based strategy for network-embedded NFP identification (Part B).

In order to evaluate the utility of the MiNeGS approach, we have compared it with a classical GS model in which all genes are treated individually. We have also evaluated the relative individual contributions of the two new aspects of the MiNeGS model by comparing MiNeGS with a microRNA-integrated GS model (MiGS; Parts A and C) and a network-embedded GS model (NeGS; Parts B and C). In the MiGS model, only the microRNA-integrated strategy is used to modify the classic GS. For a given a gene *g*, MiGS simply selects the highest-correlated k genes (k = 3 for consistency with MiNeGS) from *g*'s first-order neighbors (i.e., those directly linked to *g* in a given network). In the NeGS model, gene-gene correlations are estimated solely using microarray mRNA expression data. Comparisons between MiGS and MiNeGS reveal the benefits of a diffusion-distance-based NFP identification strategy, while comparisons between NeGS and MiNeGS indicate the contributions of integrating microRNA information with microarray data.

We used these models to analyze a diabetes microarray dataset. This dataset, published by Mootha et al [23], records the expression profiles of 22,000 genes in skeletal muscle biopsies of 43 age-matched men – 17 with normal glucose tolerance (NGT), 8 with impaired glucose tolerance (IGT) and 18 with type 2 diabetes mellitus (DM2). We used the 17 NGT and 18 DM2 samples from this dataset in order to focus our analysis on the detection of DM2-associated genes.

### B. MiNeGS identifies functionally coordinated genes

We first evaluated the NFP-identification performance of MiNeGS. Since most genes act within a single functional pathway, rather than across different pathways (i.e., do not play a role in crosstalk between pathways), we identified the 3 closest functional gene partners for each gene as its NFPs. It thus could be expected that genes and their NFPs are located within the same functional pathway, sharing common GO annotations or pathway assignments. With this idea, we estimated the functional coordination status of each gene and its NFPs. We considered a given gene *g* and its three NFPs (assuming that all of them have been annotated with a GO term or pathway assignment) to be functionally coordinated when at least 3 of them could be placed into common GO categories or pathways. In order to avoid erroneously estimates, we discarded large GO annotation categories and pathways (>200 genes) during our coordination analysis.

The coordination rates of MiGS (43%) and MiNeGS (42%) are better, albeit not remarkably so, than that of NeGS (40%). These comparisons suggest that microRNA:gene target information can improve NFP identification. NFP coordination rates of MiGS and MiNeGS are similar, but MiGS only considers first-order network neighbors and is thus unable to assign NFPs to genes that have fewer than 3 first-order network connections in our study, while MiNeGS, which is not limited to first-order connections, can assign NFPs to all given genes. Due to the scale-free nature of gene networks and limitations on the ability to computationally detect gene interactions, a large number of genes have few detected connections. In our study, 52% of the data set (1029 genes) had fewer than 3 first-order neighbors and could thus not be analyzed by MiGS. Under such situations, MiNeGS exploits more network information than MiGS, and MiNeGS outperforms MiGS in terms of GS, as indicated later.

**Figure 2:**
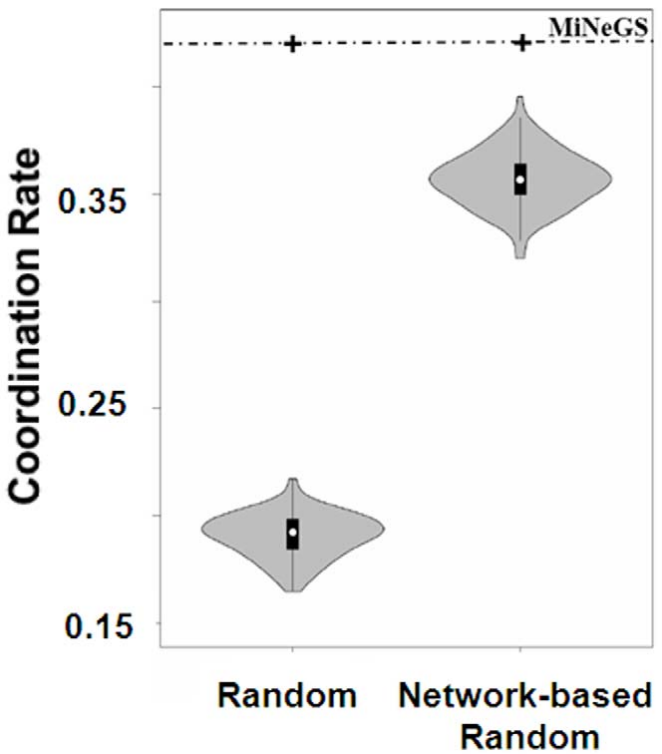
MiNeGS can identify functional coordinated NFPs with high significance.

Built-in factors of datasets may influence coordination rates. We therefore performed two additional analyses in order to determine the potential contribution of built-in dataset factors. First, to analyze the possible contributions of GO annotations and pathway assignments, we generated NFPs by randomly selecting 3 genes as the NFPs for a given gene and determined the coordination rate on the entire gene set. After repeating this process 100 times, the coordination rate distribution of the randomized NFPs was estimated. Second, to evaluate the influence of network topology, we generated network-based random NFPs for each gene by randomly selecting 3 genes from its first order network neighbors. After repeating this process 100 times we established the coordination rate distribution of the network-based random NFPs. Coordination rates derived from these two randomization processes, depicted as violin plots in Fig. 2, are significantly lower than that of MiNeGS (one sample t-test *p*-values were estimated as 4×10^−136^ when compared with the randomized NFPs and as 3×10^−71^ when compared with network-based randomized NFPs). With these almost-zero *p*-values, we can conclude that built-in dataset factors are not responsible for the coordination rate achieved by MiNeGS and, thus, that MiNeGS is able to effectively identify functional NFPs.

### C. MiNeGS increases classification accuracy

**Figure 3:**
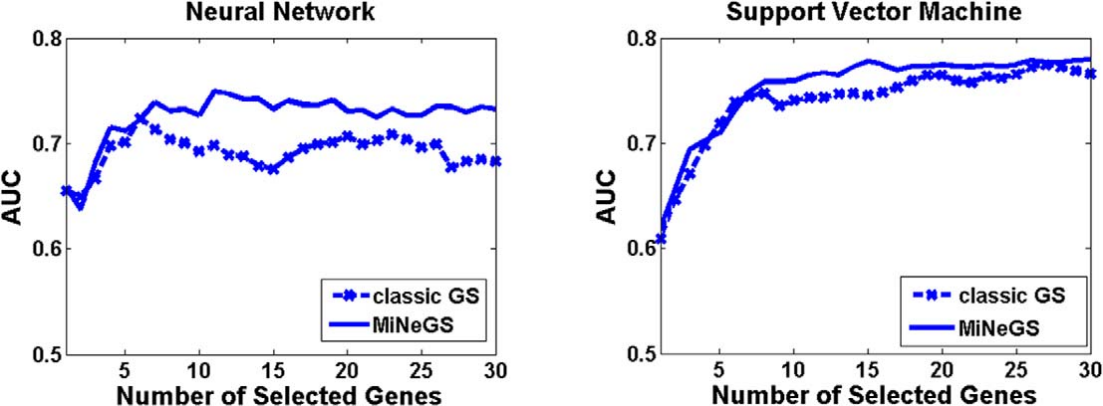
Comparisons of the classification performance of classic GS and MiNeGS approaches, using ROC AUC to measure classification performance.

The utility of a GS model ultimately rests in its classification performance, the ability to identify genes that can distinct the samples with different phenotypes. When the genes selected by a GS model enable classifiers to achieve high classification accuracy, the tested GS model can be regarded as good. We therefore examined the performance of MiNeGS by the corresponding classification results. We employed two typical classifiers, i.e., neural networks and support vector machine, for our evaluation. In order to avoid the “information leakage” and the randomness caused by partitioning analysis samples into training and testing groups, we adopted a cross validation scheme, and independently ran this scheme 20 times. In order to make comprehensive comparisons, we used each model to produce a series of gene subsets, containing from 1 to 30 selected genes, and measured classification performance by the area under the receiver operating characteristic curve (AUC), as detailed in Materials and Methods. Results of these analyses are shown in Fig. 3. Large AUCs indicate good classification performance, and thus correspond to a promising GS model. MiNeGS AUCs were generally better than those achieved by the classic GS model when using either neural networks (NN) or support vector machine (SVM) classifiers. With the classification improvements, we conclude that the effective use of microRNA target and gene network information can enhance the performance of GS. Also, in our example, SVM basically outperforms NN in that, using the classic GS model, AUCs reached by NNs is less than 0.7 at almost all cases, while SVMs can arrive AUC>0.75 in the most cases when the number of selected genes >7. As such the improvements measured by SVM AUC are thus not as remarkable as by NN AUC.

### D. MiNeGS detects more DM2-associated genes than other GS methods

DM2 has been actively studied for decades, resulting in the accumulation of a large body of biological and clinical results that can be explored to evaluate the performance of our GS models. We assemble a list of DM2-associated genes from multiple sources, a recently published article [24] and three public databases. The public databases are T2D-Db (available at http://t2ddb.ibab.ac.in/home.shtml), HuGe Navigator [25] and the curated database of the Ingenuity Pathway Analysis software. Based on information from these sources, we identified 119 DM2-associated genes from our gene set, and used them as “hallmark” genes to evaluate the performance of our GS model. We considered the inclusion of these hallmark genes in a GS result as an indicator of appropriate GS, and that increases in the number of these hallmark genes indicated improved GS performance.

**Figure 4:**
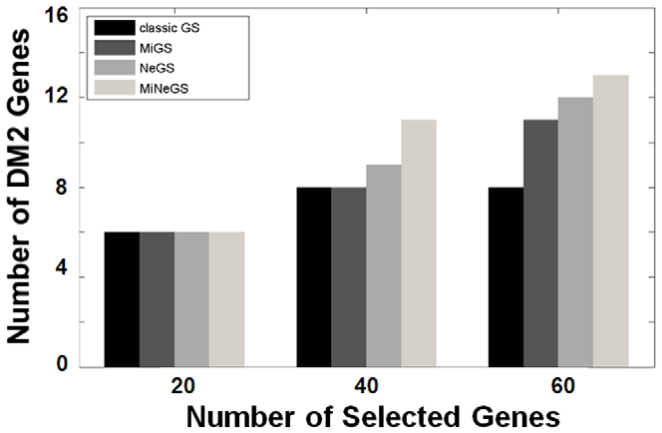
Evaluations based on the DM2 hallmark genes.

As shown in Fig. 4, all MiGS, NeGS and MiNeGS identified more DM2 hallmark genes than classical GS when they were used to select relatively large gene sets. MiNeGS identify the most hallmark genes, followed by NeGS and MiGS. This suggests that inclusion of microRNA and network information can improve the performance of GS. MiGS and NeGS address different information and modify the GS model from different perspectives. NeGS performs modestly better than MiGS, possibly due to 1) modest or variable miRNA regulatory effects [26] and/or 2) potential noise contamination of the currently available microRNA binding information [16]. However, despite these potential problems, microRNA information retains selective power since more hallmark genes can be detected out when MiGS is compared with the classic GS, and MiNeGS is compared with NeGS.

Table 1 lists the first 20 genes selected by MiNeGS. Of these genes, some play functions in energy metabolism and inflammation, the processes known to have close DM2 associations. Also, among these 20 genes, 6 are hallmark genes. Several of the remaining genes, moreover, are either associated with DM2-related conditions, such as obesity, or are supported by recently published evidence. For example, F13A1 has been linked to two DM2 related diseases, obesity and inflammation, according to the HuGe knowledge base. The protein encoded by DYNLL1 is part of the cytoplasmic dynein motor, which can regulate glucose-induced insulin secretion [27]. Several of the remaining genes have not been well studied, having few or no references in the HuGe or NCBI GeneRIF databases, so that it is difficult to evaluate their potential DM2 association.

**Table 1:** Top 20 MiNeGS selected genes.

Gene Name	Rank	DM2 hallmark	GeneRIF	HuGe
*Energy metabolism*				
PFKFB1	2	Yes	1	0
FH	3	Yes	28	8
PDK4	6	Yes	14	1
NDUFA10	7	Yes	0	2
*Cytoskeleton and cell motility*				
TCAP	9	No	11	0
DYSF	10	No	35	0
FLNC	11	No	11	0
ACTN2	12	No	5	1
MYH7	13	No	32	11
PLS3	14	No	6	0
GSN	15	No	47	0
*Inflammation*				
EGFR	16	Yes	1013	65
AGE-R1	17	Yes	0	0
*Others*				
SLA	1	No	1	0
ADSL	4	No	8	0
TRIM38	5	No	0	0
EIF3S9	8	No	1	0
ZNF207	18	No	0	0
F13A1	19	No	63	81
DYNLL1	20	No	12	0

The numbers listed in the last two columns indicate, respectively, the number of functional annotations and diseases linked to the corresponding genes in the Entrez Gene database and HuGE Navigator knowledge base.

### E. Genes selected by MiNeGS are involved in DM2 related pathways/processes

Finally, we examined the biological significance of gene selection results through comparing selected genes with functional gene categories. These categories, generally defined based on biological evidence, consist of genes that exert the same or related functional roles, participate in the same signaling or metabolic pathways, or are located within the same cytogenetic band. We organized functional gene subsets based on gene annotation and pathway information. We downloaded gene annotations from Gene ontology database (http://www.geneontology.org/), and pathway information from two databases, the molecular signature database (MSigDB) and the pathway interaction database (PID). MsigDB (available at http://www.broad.mit.edu/gsea/msigdb/index.jsp), compiles information from multiple different sites (BioCarta, KEGG, GenMAPP, etc.), while the PID database (http://pid.nci.nih.gov/) is curated by editors of the Nature Publishing Group and reviewed by experts in the field. The above databases result to 4152 distinct functional gene subsets, 3404 of which are derived from GO categories and the rest from the pathway databases.

We used the DM2 hallmark genes (as introduced in the last section) to identify DM2-associated functional gene subsets in a statistic way. Briefly, for each functional gene subset, its enrichment p-value was estimated under a hyper-geometric statistics framework. After that, all enrichment p-values were corrected according to the multiple hypotheses testing scheme proposed by Benjamini et al [28] to control the false-positive rate of this analysis. Small (<10 genes) and large (>200 genes) functional gene subsets were excluded in order to achieve reliable enrichment estimations. Based on these initial parameters and using a corrected *p*-value of p<0.1 as the threshold for statistical significance, we detected 60 DM2-associated functional gene subsets, representing 34 GO categories and 26 pathways (5 from PID and 21 from MsigDB). The majority of these gene subsets represent processes with well-established DM2 associations (such as, glucose metabolism, peroxisome proliferator-activated receptor (PPAR) signaling, insulin signaling, lipid metabolism, etc.), while a few are fundamental or essential biological processes (for instance, MAPK signaling, cell adherance, and molecular transport, etc.).

**Figure 5:**
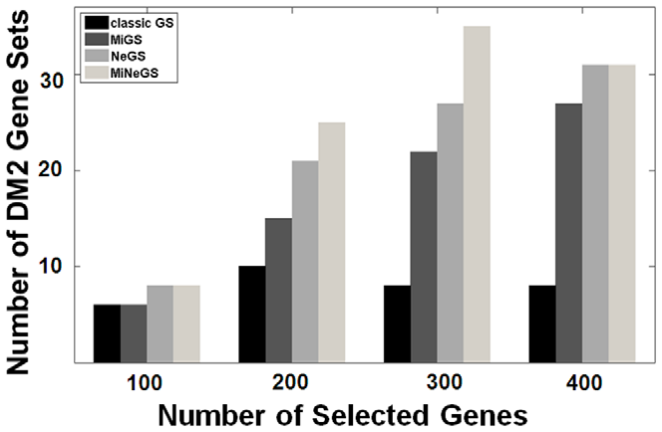
Comparisons in terms of biological meaning. The numbers of DM2 gene sets identified by different methods are compared.

We compared each GS result against all DM2-associated genes in the way mentioned in the last paragraph, i.e., using hypergeometric distribution-based significance enrichment evaluation with Benjamini false-discovery rate correction, to detect functional gene subsets enriched by the selection. The relative selective performance of four different GS models is presented in Fig. 5, with more selected DM2-associated subsets indicating better selective performance. All four methods produce similar results when used to select 100 genes. Note, however, that with increasing numbers of selected genes all three of the modified GS models (MiGS, NeGS and MiNeGS) identify more DM2-associated gene sets than the classical GS model, whose identification rate does not change. MiNeGS identified the most DM2-associated gene subsets when 200 or 300 genes were selected by each model, and tied with the NeGS model upon selection of 100 or 400 genes.

MiNeGS detected 35 DM2-associated functional subsets, with a *p*-value<0.1, when used to select 300 genes. The subsets with the highest significance (p-value<0.01) amongst these 35 subsets are listed in Table 2. This list covers a number of DM2-assocated processes or functions, such as fatty acid oxidation, hormone activity, insulin signaling, and glucose metabolism, among others. Based on these results, MiNeGS offers a clear advantage over classical GS in the identification of functional gene sets when used to select increasing numbers of genes, and performs as well or better than NeGS when used to select any number of genes.

**Table 2:** Gene subsets enriched by highly significant (corrected *p* value<0.01) MiNeGS genes.

	Functional Gene Subset
1	GO:0006635 fatty acid beta-oxidation process
2	GO:0005179 hormone activity function
3	GO:0046982 protein heterodimerization activity function
4	GO:0008286 insulin receptor signaling pathway
5	GO:0004871 signal transducer activity function
6	GO:0006006 glucose metabolic process
7	KEGG IL4 receptor in B lypthocytes
8	KEGG Type II diabetes mellitus
9	PID insulin pathway
10	PID the ptp1b-mediated signaling pathway
11	PID the txa2-medicated signaling pathway

### F. Conclusions

Microarray data analysis for the selection of candidate genes is often the crucial step in the analysis of the molecular mechanisms of complex biological phenomena and diseases. Gene network information has been incorporated into microarray-based GS studies to reflect the fact that a gene may interact with several other genes to exert its phenotype.

In this paper, we present a novel network-embedded GS model. In a network-embedded model, identifying gene functional partners is the crucial aspect. To effectively cope with this issue, we address two challenges and make contributions from two perspectives. First, in order to improve upon the limitations of microarray data, we now propose a method to combine microarray expression data with mRNA:microRNA binding information. This approach attempts to account for microRNA posttranscriptional effects on mRNA levels in order to better estimate the ultimate expression of a given gene. Second, we introduce the concept of diffusion distance to estimate the degree of interaction of all gene pairs. With this concept, we can include network connections, and take into account all possible paths connecting gene pairs. These capabilities are needed to reflect the complicated mechanism of gene interaction that can occur in even simple biological systems.

We have evaluated these proposed strategies from both computational and biological perspectives. Our results show that an approach that integrates microRNA binding data with expression data can enhance the identification performance of functional partners of genes. More importantly, comparison of MiNeGS and it component methods with classical GS methodology clearly shows the advantages of the proposed strategies since, after applying these strategies, GS performance is enhanced and selected genes demonstrate higher classification capabilities. MiNeGS selected more previously-reported disease genes than the classic GS model when these methods were used to analyze a DM2 data set. Gene-set-enrichment analysis results also showed that the MiNeGS gene lists contained many well-known or newly-confirmed DM2-associated processes and pathways. In summary, these results suggest that GS results from the MiNeGS method provide high-quality, and functionally relevant biological information, in this case showing promise to identify genes involved in DM2.

## Materials and Methods

### A. Data

In order to evaluate our GS models we used a previously published microarray data [23] which was derived from the skeletal muscle biopsies of 43 age-matched men with normal glucose tolerance (NGT, number of samples = 17), impaired glucose tolerance (IGT, N = 8), or diabetes type II (DM2, N = 18). We omitted all IGT subject data to compare DM2-associated changes between NGT and DM2 groups. Similar to the original study by Mootha et. al. [23], we deleted genes that demonstrated low expression over the entire data set. Genes with expression values <100 were considered to be unexpressed, and genes whose average expression were <100 were excluded from our analysis. Genes with low variation (standard deviations <0.2) across samples were also excluded from analysis, since, for these genes, experimental noise may markedly contribute to variation.

The microRNA:gene target and gene network data used in our models downloaded from public databases. We download experimentally-verified microRNA targets from TarBase (http://diana.cslab.ece.ntua.gr/tarbase/) and computationally predicted microRNA target results from PicTar (http://pictar.mdc-berlin.de/) and TargetScan (http://www.targetscan.org/). Due to the scarcity of experimentally-verified results, microRNA:target assignments are largely derived from computational predictions. Most existing prediction models, such as TargetScan, miRanda, PicTar, RNA22, etc, adopt similar principles (the microRNA binding sites must match to the seeds of microRNAs in a Watson-Crick pairing way and must be evolutionarily conserved) and differ only in their technical details [16]. Also in order to control false positive rate of prediction, we concentrated on the methods that use stringent seed-matching criteria and consider the evolutionary conservation of binding sites [34]. Two well-developed tools, PicTar and Targetscan, were thus considered in our study. A microRNA is considered to bind an mRNA when this relationship is supported by experimental evidence, or predicted by either PicTar or Targetscan. Finally, microRNAs that have <100 targets were considered not to be well-studied and were excluded from this study.

Following other studies [11], [12], [29], we used protein-protein interaction databases to build our network. Here we explored information provided by STRING (search tool for the retrieval of interacting genes/proteins). STRING is a comprehensive database, collecting the interactions from well-known resources, such as MINT, HPRD, BIND, DIP, BioGRID, KEGG, NCI-Nature Pathway Interaction, among others [30]. The interactions provided in STRING cover a wide spectrum of cell types and environmental conditions, and a part of them work in our studied scenario. To exclude irrelevant interactions, we eliminated those having negative mRNA-based correlations.

After filtering out the genes having small variations and no interactions with others, and the microRNAs having sparse binding targets, we finally arrived at 1977 genes and 489 microRNAs. Among those genes, 1338 (67%) are (potentially) bound by microRNAs. Also, we have up to 80,000 interactions. The distribution of connection degrees of genes follows a power law – 637 genes (32%) have less-than 10 connections, while only 24 genes (4.3%) have more-than-150 ones.

### B. Methods

Our system, as overviewed in Fig. 1, includes three components (A, B and C), and uses as input microarray expression data, microRNA binding composition data and *a priori* known gene networks. Through integrating the first two types of data, the correlations between genes are evaluated. Then after overlaying those gene correlation estimates onto gene networks, by using the concept of diffusion distance, the nearest functional partners (NFPs) of each gene are detected. Finally, based on microarray expression data and NFP identifications, the gene-phenotype association is estimated for each gene, and the genes having high associations are naturally marked out as biomarker gene. Our main contributions lie in the part A and B.

Suppose we have a gene expression dataset *D* = {*X*, *Y*}. *X* = [***x*** *_1_*,…***x*** *_j_*,…,***x*** *_n_*] records the transcriptional profiles having *m* genes for *n* samples, and ***x*** *_i_* = [*x_i1_*,…*x_ij_*,…,*x_in_*]*^T^* (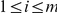) where *x_ij_* is the transcriptional expression of the *i*th gene in the *j*th sample. *Y* = [***y*** *_1_*,…***y*** *_j_*,…,***y*** *_n_*] where ***y*** *_j_* indicates the phenotypic responses of the *j*th samples. Also the potential binding relationships between *m* genes and *k* microRNAs are collected in a binary matrix *Z* = [*z_iq_*]*_m×k_*, where *z_iq_* = 1 indicates that the *i*th gene can be hybridized and then regulated by the *q*th microRNA based on experimental or computational prediction results, otherwise *z_iq_* = 0. To construct *Z*, we combine the results of biological experiments and well-developed microRNA target predication tools, as detailed in the last section. Finally, we have a graph Ω = {*V*, *E*} to reflect a prior known gene networks. The vertex set *V* is our gene set, covering all studied *m* genes. The edge matrix *E* = [*e_ij_*]*_m×m_*. When the *i*th gene and the *j*th are connected together based on known gene networks, we have *e_ij_* = 1; otherwise *e_ij_* = 0.

#### B.1 MicroRNA-integrated Gene Correlation

In order to search NFPs of genes, estimating gene correlation is the first step. For this purpose, we consider two factors – one is the transcriptional expressions of genes, which are given in microarray gene expression data, and the other is the potential post-transcriptional effects on gene expression, which is critical in certain cases and which we believe has not received enough emphasis in current GS studies.

Given two genes (say, the *i*th and *j*th genes in our data), their transcriptional expressions are stored in ***x*** *_i_* and ***x*** *_j_*, and their microRNA binding compositions are described by ***z*** *_i_* and ***z*** *_j_*. To evaluate the correlation between ***x*** *_i_* and ***x*** *_j_*, we use Pearson correlation coefficient (*PCC*). As to microRNA-binding composition binary variables, we explore the concept of Jaccard similarity coefficient. Jaccard similarity coefficient (*J*), measuring the percentage of nonzero coordinates that are same, is defined in a way of
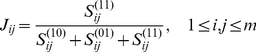
(1)where 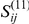 is the number of *r* satisfying *z_ir_* = *z_jr_* = 1. Similarly, 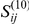 is the number of *r* with *z_ir_* = 1 and *z_jr_* = 0, while 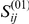 is for *z_ir_* = 0 and *z_jr_* = 1. A large *J*_ij_ indicates that two genes are regulated by much like microRNA sets. *J*_ij_ arrives its maximum 1 when two corresponding genes are regulated by the same group of microRNAs. Moreover, we have *J* = 1 between two non-microRNA-binding genes, while *J* = 0 between a microRNA-binding and a non-microRNA-binding gene.

Combining the Pearson correlation coefficient *PCC*_ij_ and Jaccard similarity coefficient *J*_ij_ together, we finally have the correlation between the *i*th and the *j*th gene as
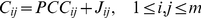
(2)


#### B.2 Network-embedded Functional Nearest Partners based on Diffusion Map

It has been reported that the sensitivity and specificity of GS can be enhanced by inclusion of gene network data. Our study examines the capability of a gene not only based on its expression but also on the expression of its working partners. For each gene, we detect its nearest functional partners (NFPs). The idea behind our NFP identification is that, in order to work together, a gene (say, *g_i_*) and its NFPs must have highly-correlated expressions, and at the same time belong to the same pathway.

Given a gene network, two genes are first-order neighbor to each other when they have a direct connection. Two genes are high-order neighbors to each other when they are connected through other genes. Based on the gene network and our gene correlation estimates (eq. 2), the straightforward way to identify the NFPs of *g_i_* is to sort out the genes having top expressional correlations to *g_i_*, among all the first-order neighbors of *g_i_*. Focusing on first-order neighbors is not enough when it is known that, through the propagation of biological signals, a gene may interplay with its high-order neighbors. In order to include high-order neighbors for functional neighbor examination, one may consider the concept of shortest path in the graphic theory and measure the distance of two genes as the length of the shortest path between them. The problem underlying this idea is that, in complicate gene networks, two genes may be connected through different paths, and two genes may closely collaborate when they are modestly connected through many paths in a gene network. As such, the shortest path is not comprehensive enough to capture the information of all the paths connecting two genes. To this end, we resort to diffusion distance and diffusion maps to detect NFPs. Diffusion distance is defined to measure the distance between graph nodes with considerations of the information of high-order neighborhood, whilst diffusion maps allows us to evaluate diffusion distance in an effective way.

Diffusion distance was first proposed by Coinfman et al. [22] and has been used for dimensionality reduction and graph-based data clustering [31], [32]. The final goal of diffusion-distance-based methodologies is to reduce the complexity of a given dataset while retaining original geometry as much as possible. To achieve this goal, a finite graph is firstly constructed to accurately reflect geometric relations among given data points. In our example, such graph is built through weighting the edges of the gene network Ω with gene correlation estimates. In this correlation-weighted graph, the vertices are all studied genes, while the edge matrix, below denoted as 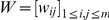, is determined by
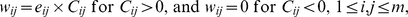
where *e_ij_* is an edge of Ω. *e_ij_* is 1 when the *i*th and *j*th genes are connected in the network Ω; otherwise *e_ij_* = 0. *W* is symmetric since 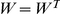, i.e., 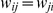. Also, *W* is pointwise positive because of 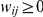 (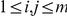). Further, a diagonal matrix *D* is built so that its diagonal element *d_jj_* (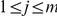) is the sum of similarities of the *j*th gene to all genes, i.e., 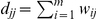. Normalizing *W* with *D*, we arrive at 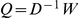. Clearly, we have 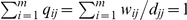, i.e., the sum of elements in a column of *Q* is 1. With this observation, *Q* can be seen as a Markov random transition matrix, and *q*_ij_ is thus interpreted as the probability of transition from the *i*th to the *j*th gene in one time step. Instatistical nomenclature, 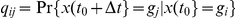, where *t_0_* and 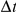 are the initial time and a time step, respectively. By *Q*, the first-order neighbor closeness between genes is explicitly reflected. Furthermore, *Q^2^* is a Markov transition matrix since 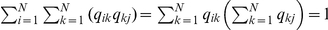. An element in *Q^2^* (below denoted as 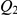 for the sake of convenience) can be regarded the probability of one gene to another in two random walk steps. For example, we have 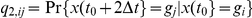. Along this reasoning direction, an element in 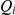 gives us the probability of signal propagating from one gene onto another after *l* random walking steps. The above analysis shows that, by taking powers of *Q*, the signals are diffused forward in the gene networks, and the elements in *Q_l_* reflect gene similarity after considering high-order neighborhood. Below, for simplification, we set *t_0_* = 0, and denote 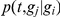 and *Q*_t_ respectively as 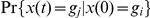 and the transition matrix at the *t*th time unit.

Based on the matrix *Q_t_*, the next step is to measure the distance between graph nodes. Given a graph, two nodes will be close if they are connected through several paths. The more paths two nodes are connected by, the closer they may be. To reflect this fact, the diffusion distance at the time *t* is defined as the weighted Euclidean distance, i.e.,
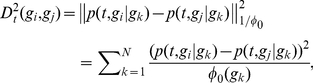
(3)where 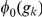 is the normalized degree of *g_k_*, i.e., 
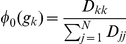
. With 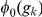
(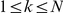), the diffusion distance (eq. 3) takes into account the empirical local density of graph nodes (i.e., genes in our example), giving the emphasis on the low-density nodes and avoiding the dominant of the high-density ones.

Following the studies in the spectral clustering, the eigenvectors of the transit matrix *Q_t_* are used to map the original space to a new space where the clusters of the points are presented in a clearer way than in the original space. Our *m*-dimensional Markov transition matrix *Q* has *m* eigenvalues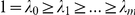, with the corresponding eigenvectors {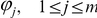} that satisfy 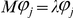. Using the top rescaled *L* eigenvectors as a new set of coordinates, the original genes (represented by the correlation-weighted graph) are mapped to an *L*-dimensional Euclidean space by
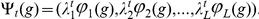
Such a process is called diffusion mapping. As proved by Nadler et al. [33], the diffusion distance (eq. 3) can be accurately approximated by the Euclidean distance in the new space, i.e.,
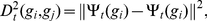
(4)


The diffusion distance (eqs. 3–4) negatively reflects the collaboration relationship between genes. Based on this index, for a given gene, we detect out its *k* nearest neighbors. After placing gene correlation estimation onto gene networks, we construct a weighted graph whose nodes cover all genes, and whose edges reflect the first-order collaboration probability between genes. Upon this graph, the Markov matrix *Q_t_* makes us simulate the situation of biological signal propagation, reflecting interplay between genes, either directly or indirectly connected. With *Q_t_*, the diffusion distance (eqs. 3–4) enables us to follow the biological nature that genes may collaborate through the multi-paths to estimate their collaboration closeness.

#### B.2 Network-embedded Gene-phenotype association

A gene always varies in a similar way with their functional partners. Based on this idea, Chuang et al. [29] averaged the normalized expression levels of the genes within a sub-network, and used this average to check whether that sub-network was associated to breast cancer metastasis or not. A gene sub-network here is a set of genes that are functional partners to each other. Rapaport et al. [11] detected working partners for each gene from its first-order-neighbors. Then, after assuming that genes have the similar expressions with their working partners, they filtered out high-frequency noise in expression data to enhance the performance of GS and classification.

Based upon microarray data, we first evaluate the correlation between genes and the phenotype response. Many indexes, such as student *t* test, Kruskal-Wallis test and mutual information (MI), can be used for this purpose. In this study, MI is employed in that MI is a flexible nonparametric tool and can measure the relationship of variables with arbitrary distributions. Given a gene (say *g_i_*, its expression variable is ***x_i_***), the MI of that gene to the discrete phenotype response variable ***y*** is defined as
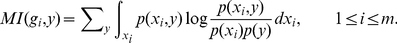
(5)We estimate MI (eq. 5) in a popularly-used way where the space of *x_i_* is equally divided into 10 bins at first and histograms for *p*(*x*,*y*) and *p*(*x*) are then built.

Based on MI estimates and NFP identifications, we determine the gene-phenotype association (GPA) as the average of MIs of genes and their NFPs. That is, for the gene *g_i_*, we have
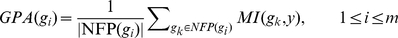
where |*a*| denotes the number of the items in *a*. Based on *GPA* estimates, the genes are finally ranked in terms of association.

### C. Classification Scheme

GS models are evaluated in terms of classification performance. A GS model is firstly required to select *J* genes. Then by using those *J* genes, two typical classification models are built. Good performance of the built classifiers naturally implies a good gene subset, or equivalently, a promising GS model.

To avoid “information leakage”, we adopted a cross validation scheme. The 35 samples of our dataset were partitioned into 6 groups, each containing 6 samples (3 DM2 and 3 NGT) except for the last group that has 5 samples (2 NGT and 3 DM2). For each run, five data groups are used for GS and classifier training, while the remained group is used for a classification test. This process is repeated 6 times, with each of the 6 groups used in turn. After that all the testing results are summarized together. In order to minimize random variations introduced by data-partitioning, we independently ran the cross-validation process 20 times, and averaged the results of these trials.

Two classification models are used in our study. They are neural networks and support vector machine, which are available at NetLab (http://www.ncrg.aston.ac.uk/netlab/index.php) and the University of Southhampton ISIS website (http://www.isis.ecs.soton.ac.uk/resources/svminfo/), respectively. The number of hidden neurons of neural networks are set as ⌊*J* ^1/2^⌋, where ⌊*a*⌋ is denoted as the integral part of the value *a* and *J* is the number of the genes we use for classification. For support vector machines, we use radial base functions as their kernels. Other parameters and initial values for our classifiers are set by the model defaults.
